# EyeDiseases: an integrated resource for dedicating to genetic variants, gene expression and epigenetic factors of human eye diseases

**DOI:** 10.1093/nargab/lqab050

**Published:** 2021-06-01

**Authors:** Jian Yuan, Fukun Chen, Dandan Fan, Qi Jiang, Zhengbo Xue, Ji Zhang, Xiangyi Yu, Kai Li, Jia Qu, Jianzhong Su

**Affiliations:** School of Ophthalmology & Optometry and Eye Hospital, Wenzhou Medical University, Wenzhou 325027, China; National Clinical Research Center for Ocular Disease, Wenzhou 325027, China; Institute of Biomedical Big Data, Wenzhou Medical University, Wenzhou 325027, China; School of Ophthalmology & Optometry and Eye Hospital, Wenzhou Medical University, Wenzhou 325027, China; National Clinical Research Center for Ocular Disease, Wenzhou 325027, China; Institute of Biomedical Big Data, Wenzhou Medical University, Wenzhou 325027, China; School of Ophthalmology & Optometry and Eye Hospital, Wenzhou Medical University, Wenzhou 325027, China; National Clinical Research Center for Ocular Disease, Wenzhou 325027, China; Institute of Biomedical Big Data, Wenzhou Medical University, Wenzhou 325027, China; School of Ophthalmology & Optometry and Eye Hospital, Wenzhou Medical University, Wenzhou 325027, China; National Clinical Research Center for Ocular Disease, Wenzhou 325027, China; Institute of Biomedical Big Data, Wenzhou Medical University, Wenzhou 325027, China; School of Ophthalmology & Optometry and Eye Hospital, Wenzhou Medical University, Wenzhou 325027, China; National Clinical Research Center for Ocular Disease, Wenzhou 325027, China; Institute of Biomedical Big Data, Wenzhou Medical University, Wenzhou 325027, China; School of Ophthalmology & Optometry and Eye Hospital, Wenzhou Medical University, Wenzhou 325027, China; National Clinical Research Center for Ocular Disease, Wenzhou 325027, China; Institute of Biomedical Big Data, Wenzhou Medical University, Wenzhou 325027, China; School of Ophthalmology & Optometry and Eye Hospital, Wenzhou Medical University, Wenzhou 325027, China; National Clinical Research Center for Ocular Disease, Wenzhou 325027, China; Institute of Biomedical Big Data, Wenzhou Medical University, Wenzhou 325027, China; Wenzhou Institute, University of Chinese Academy of Sciences, Wenzhou 325011, Zhejiang, China; School of Ophthalmology & Optometry and Eye Hospital, Wenzhou Medical University, Wenzhou 325027, China; National Clinical Research Center for Ocular Disease, Wenzhou 325027, China; School of Ophthalmology & Optometry and Eye Hospital, Wenzhou Medical University, Wenzhou 325027, China; National Clinical Research Center for Ocular Disease, Wenzhou 325027, China; Institute of Biomedical Big Data, Wenzhou Medical University, Wenzhou 325027, China

## Abstract

Eye diseases are remarkably common and encompass a large and diverse range of morbidities that affect different components of the visual system and visual function. With advances in omics technology of eye disorders, genome-scale datasets have been rapidly accumulated in genetics and epigenetics field. However, the efficient collection and comprehensive analysis of different kinds of omics data are lacking. Herein, we developed **EyeDiseases** (https://eyediseases.bio-data.cn/), the first database for multi-omics data integration and interpretation of human eyes diseases. It contains 1344 disease-associated genes with genetic variation, 1774 transcription files of bulk cell expression and single-cell RNA-seq, 105 epigenomics data across 185 kinds of human eye diseases. Using EyeDiseases, we investigated SARS-CoV-2 potential tropism in eye infection and found that the SARS-CoV-2 entry factors, ACE2 and TMPRSS2 are highly correlated with cornea and keratoconus, suggest that ocular surface cells are susceptible to infection by SARS-CoV-2. Additionally, integrating analysis of Age-related macular degeneration (AMD) GWAS loci and co-expression data revealed 9 associated genes involved in HIF-1 signaling pathway and voltage-gate potassium channel complex. The EyeDiseases provides a valuable resource for accelerating the discovery and validation of candidate loci and genes contributed to the molecular diagnosis and therapeutic vulnerabilities with various eyes diseases.

## INTRODUCTION

Eye diseases encompass a large and diverse range of morbidities that affect different components of the visual system and visual function (1). In the medium- to long-term, they could cause vision impairment including blindness, such as diabetic retinopathy, glaucoma, age-related macular degeneration and cataract (2). Globally, it is estimated that at least 2.2 billion people have a vision impairment or blindness based on recently published epidemiological data (3,4). With behavioural and lifestyle changes, and urbanization, population growth and ageing will dramatically increase the number of people with eye conditions, vision impairment and blindness in the coming decades (5–9). Meanwhile, the burden of visual impairments and eye conditions tends to be greater in low and middle-income countries and underserved populations, such as women, indigenous peoples, persons with certain kinds of disability, and in rural communities.

Genetic variation also plays important roles in eye diseases in both children and adults. Over 60% of cases of childhood blindness are caused by genetic factors, such as congenital glaucoma, ocular malformations, atrophy of the optic nerve and retinitis pigmentosa (10). Although the triggering factors for many eye diseases remain unknown, genome-wide association studies (GWAS) have identified many nucleotide polymorphisms, with some of them likely to be causal, and associated risk loci (11–16). Additionally, evidences are accumulating that epigenetic changes resulting from environmental factors may play a significant role in eye disorder, and interventions to modify these factors hold increasing promise (17–19). Together, these results indicate that either hidden factors such as epigenetics or gene-environment interactions constitute the predominant factor for eye disorder risks.

**Figure 1.**
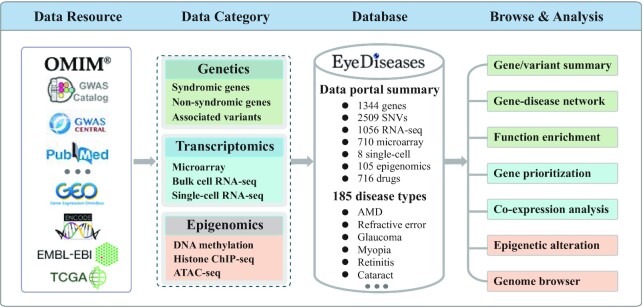
Data processing workflow and overall architecture of EyeDiseases. EyeDiseases is an omics database which integrates gene, mutation, RNA expression, Single-Cell RNAseq, DNA methylation, chromatin accessibility, histone modification and drug data. The database provides three functions, ‘Genetics’, ‘Transcriptomics’, and ‘Epigenomics’, to help researchers visualize the relationships between eye disorders and candidate genes. Data in this database were performed extended functional annotation, such as gene–disease networks, gene ontology, pathway analysis, gene prioritization, co-expression and epigenetic alteration.

Disease-specific databases are useful for researchers to find and retrieve the appropriate information. However, a gene–disease association database dedicated to eye diseases is still unavailable. With the next-generation sequencing technologies providing even more power for gene discoveries (20), it has become more and more difficult for an ophthalmologist to understand with confidence how many genes have been associated with eye diseases. In this study, we presented, Eyediseases, a manually curated database, aimed to enhance our understanding of eye diseases. EyeDiseases is a multi-omics database which integrates gene, mutation, RNA expression, Single-Cell RNA-seq, DNA methylation, chromatin accessibility, histone modification and drug datasets (Figure 1). The database provides three functions, ‘Genetics’, ‘Expression’ and ‘Epigenomics’, to help researchers visualize the relationships between eye disease and associated genes. Data in this database were performed extended functional annotation, such as gene-disease networks, gene ontology, pathway analysis, co-expression and epigenetic alteration. Therefore, EyeDiseases uses uniform processing pipelines to create high-quality, consistent, and reproducible data, which provides the scientific community with freely available information on eye disease-related gene, RNA expression and epigenetic regulation.

## MATERIALS AND METHODS

### Database structure and organization

Eyediseases integrates multi-omics data from 1138 projects. The main diseases include Refractive error, Myopia, Retinitis, Glaucoma, Cataract, Age-related macular degeneration. There are 2509 records of single nucleotide variants (SNVs) and 1344 records of genes, and the corresponding annotation are based on hg19 and hg38. There are 710 and 1056 records in the microarray and bulk cell RNA-seq expression data, respectively. The single-cell RNAseq data includes six normal eye tissues and two disease samples. There are 105 records in the epigenomics which contain DNA methylation, ATAC-seq, H3K27ac, H3K27me3, H3K4me1, H3K4me3, H3K9me3 data from 15 normal tissues and 12 disease tissues (Table 1). There are 716 records regarding variant/gene-related drug information collected from the DrugBank.

**Table 1.** Data content in Eyediseases

	Data type	Total No.
**Genetics**	Genes	1344
	Variants	2509
**Expression**	Microarray	710
	RNA-seq	1056
**Single cell**	RNA-seq	8
**Epigenomics**	ATAC-seq	7
	H3K4me1	2
	H3K4me3	4
	H3K9me3	1
	H3K27ac	5
	H3K27me3	1
	Bisulfite-Seq	2
	RRBS	3
	methylation 450	80

The EyeDiseases comprises seven modules: Search, Browse, Analysis, Statistics, Download, Document and Contact. The search module provides three modes by disease, genes or variants. The browse module contains four perspectives: disease, gene expression, single-cell transcriptomes and epigenomics. The analysis comprises five analytical perspectives: gene-disease network, functional annotations, gene prioritization, co-expression analysis and epigenetic alteration.

### Data sources and preprocessing

The disease list was obtained from the Medical Subject Headings (MeSH) (https://meshb.nlm.nih.gov/), cross-referenced with the list of diseases included in NCBI. Consequently, total 185 eye diseases were retained, which gene associations can be found by our data collection process (see below).

One important feature of EyeDiseases is comprehensive collection of data from major genetic studies for most eye disorders. For gene linked with syndromic eye disorder, we searched Online Mendelian Inheritance in Man databases (OMIM, https://omim.org/) to get the official disease names and linked all the disorders to OMIM, and searched PubMed for additional citations using the query ‘disease name’. All citations were double-checked manually. For association, we first searched and collected data with MeSH and OMIM name from the established GWAS catalog (https://www.ebi.ac.uk/gwas/) and GWAS Central (https://www.gwascentral.org/). To obtain a complete resource of multi-omics relevant to eye disorders, comprehensive searches were performed for eye studies.

We searched the GEO (https://www.ncbi.nlm.nih.gov/geo/) and EMBL-EBI (https://www.ebi.ac.uk/) database with the following query terms: ‘(eye OR disease name) AND (microarray OR transcriptome OR RNAseq)’ for expression profiling studies, ‘(eye OR disease name) AND (single cell RNA-seq OR scRNA-seq)’ for single cell profiling studies and ‘(eye OR disease name) AND (DNA methylation OR histone OR ATAC-seq)’ for epigenomic datasets. Each type of data includes normal tissues and disease tissues. We also achieved the datasets from ENCODE (https://www.encodeproject.org/), and TCGA (https://portal.gdc.cancer.gov/) database.

For the expression profile generated with the next-generation RNA-seq platform, we aligned RNA-seq reads in BAM format using a two-pass method with STAR. Following alignment, gene-level expression quantifications based on a collapsed version of a reference transcript annotation, provided as read counts and TPM. For the microarray profile, Illumina microarrays were log2 transformed and quantile normalized using the lumi package in R. Affymetrix microarrays were RMA normalized (background correction, log2 transformation, quantile normalization, and probe summarization) using the affy package in R. Agilent microarrays were normalized using the limma package in R (read.maimages, backgroundCorrect, normalizeWithinArrays, normalizeBetweenArrays, getEAWP functions). To provide a systematic nomenclature for assessment of gene expression across platforms, microarray probes were re-mapped to Ensembl gene IDs and gene symbol using the biomaRt package in R, taking the maximum mean signal across all probes available for each gene, using the collapseRows function. The collapseRows MaxMean function was explicitly developed to perform cross-platform microarray meta-analysis and has been extensively validated in its ability to increase between-study consistency and enhance reproducibility (21). Strict care was taken to ensure data integrity.

For single cell RNA-seq, we following the Seurat Clustering Tutorial (https://satijalab.org/seurat) to cluster the cells and find cell markers that define clusters via differential expression (22). DNA methylation, histone ChIP-seq and ATAC-seq analysis were performed by the ENCODE and TCGA data coordinating center uniform processing pipelines to create high-quality, consistent, and reproducible data (23). We also developed a user-friendly and configurable genome browser through which multiple genomic and epigenomic resources can be visualized simultaneously.

### Data analysis

In order to better understanding the phenotypic and genetic complexity in eye diseases, gene-disease network developed by D3.js was constructed to demonstrate the overlapping features between diseases. Next, enriched gene ontology, including biological process, cellular component, molecular function and functional pathways, were identified by DAVID. For each gene, we computed and displayed its co-expressed genes by weighted gene co-expression network analysis (WGCNA) in disease and normal samples, respectively. We used the soft threshold method for pearson correlation analysis of the expression profiles to determine the connection strengths between two genes. To select and prioritize candidate genes for complex eye diseases, we calculated the gene significance (GS) of a gene as the Pearson correlation coefficient for testing the association of the gene expression to the disease type. Generally, if GS were highly associated, it implied that genes were most significantly correlated with the disease. Furthermore, to systematic characterize candidate causal genes for AMD, we observed the assay for transposase-accessible chromatin with sequencing (ATAC-Seq) peak in AMD associated genes and find the extent to which epigenetic changes regulate AMD.

### Implementation

EyeDiseases codes were developed using an integrated development environment, the server is implemented based on the Flask (https://flask.palletsprojects.com) framework. PostgreSQL (12–12.1–2) is used to store and manage the data information of the database. EyeDiseases uses Vue (2.6.11) for most of its front-end components and adopts. The UI that is compatible with PC and mobile terminals is completed through Bulma (https://bulma.zcopy.site). All drawing functions in the EyeDiseases are realized by using D3 (5.16.0) and Plotly (http://chart-studio.plotly.com), The tables of the database are visualized using ag-gird. The entire web application is deployed on CentOS (one of the most popular Linux distribution; https://www.centos.org) operating system. All the data and code used is publicly available at https://github.com/sulab-wmu/eyediseases.

## RESULTS

### Database interface

We developed a user-friendly web interface for Eyediseases. The user may access all data and conduct analysis through the web interface (https://eyediseases.bio-data.cn/). To support facilitating access to genetic and epigenetic data in Eyediseases, five main search and explore approaches have been provided in home page: search by disease, search by gene, search by variant, explore by transcriptomics and explore by epigenomics (Figure 1). For disease query, we provide widgets that allow users to search for diseases by user supplied keywords (e.g. age-related macular degeneration). After clicking on a disease name, users will be redirected to a new page to choose data types. Once the user clicks genetics module, a detailed gene and variant page is shown, includes a list of associated genes and evidence supporting the association. For genetics query, users can find a quick search box on the home page to search by gene symbol (e.g. CFH) or variant (e.g. rs10490924). The result page for single gene search has five major views: (i) summary view provides an overview of general information of the queried gene; (ii) disease and phenotype view provides an associated disease and phenotype information from HPO; (iii) external reference view provides web link to the related databases when available; (iv) drug views provides a suite of a drug interacts with queried gene powered by the DrugBank platform and (v) expression view displays the expression patterns of a specific gene in the archived transcriptome datasets. The ‘variant search interface’ allows the user to input a single variant site (e.g. rs10490924), and return results for the collected mutation information list and the population frequency of the mutation. In disease and genetics query, all data sets are relationally linked. Once the user clicks a gene symbol or rs ID, a detail gene or variant page is shown.

In the transcriptomics module, the user may browse expression data by (i) clicking one of the three data categories (‘Multi Gene Query’, ‘Eye Development’ and ‘Single-Cell RNAseq’) on the home page; or (ii) selecting expression or single-cell RNAseq of ‘Browse’ on the function bar on the top of the web page. The expression search section, multi-gene query visualization allows users to select platform, species and datasets, and enter a list of genes of interest.

Next, users can click submit to view the expression of those genes in a table and a heat map that shows expression level across all normal or disease tissues. By default, the genes and tissues are clustered using hierarchical clustering. You can download the expression matrix and figure for further analysis. In the single-cell RNA-seq section, the results are showed by project. The view shows t-SNE plots of all cell type as well as a computed matrix table of cell markers with columns (i) cluster ID, (ii) cell type, (iii) cell marker, (iv) *P*-value, (v) average log_2_ fold change; (v) PC1 and (vi) PC2. In the Epigenomics view, Eyediseases provides a user-friendly and configurable genome browser through which multiple genomic and epigenomic resources can be visualized simultaneously. The genomic viewer connecting to a PostgreSQL backend is used to show the landscape for normal and disease tissue epigenomic information. Features of the viewer include the ability to zoom through the given regions, to enter a region by specifying the genomic coordinates, to change the order of tracks and to show and hide certain feature tracks and configure the appearance of the displayed information.

### Example usage 1: EyeDiseases is used to find susceptible tissues in SARS-CoV-2 infection

COVID-19 runs rampant around the world (24). Ocular signs and symptoms are observed in a subset of patients with COVID-19, including conjunctival hyperemia, chemosis, epiphora, increased secretions, ocular pain, photophobia and dry eye (25). Recently, immunohistochemical studies show the ocular surface localisation of SARS-CoV-2 entry factors, ACE receptor and TMPRSS2 protease in human eyes (26,27). We performed association analysis of individual genes with the eye tissues and diseases by WGCNA co-expression network analysis. Using ‘gene significance’ module in ‘analysis’ page of database, we searched seven susceptibility gene, including ACE and TMPRSS2, to view the gene-tissue/disease association score in a table. We found that ACE2 and TMPRSS2 expression level were highly correlated with corneas and keratoconus (Table 2). This result indicated that corneal cells are susceptible to infection by SARS-CoV-2 and eye protection is advisable to prevent contamination from contact lenses, external droplets and hand-eye contact.

**Table 2.** Gene significance (GS) score of SARS-CoV-2 entry factors and susceptibility gene in disease and normal tissues

Normal	ACE2	TMPRSS2	CCR9	FYCO1	CXCR6	XCR1	SLC6A20	LZTFL1
cornea	0.364	0.300	0.032	−0.425	0.022	−0.017	−0.090	−0.258
retina	−0.119	−0.155	−0.264	0.225	−0.254	−0.192	−0.301	0.305
retina_macula	−0.138	−0.137	−0.146	0.086	−0.174	−0.162	−0.255	0.311
retina_non_macula	−0.144	−0.134	−0.146	0.292	−0.195	−0.176	−0.265	0.424
rpe_macula	0.305	−0.016	0.078	−0.073	0.458	0.222	0.319	−0.410
rpe_non_macula	−0.037	0.120	0.396	−0.120	0.243	0.263	0.609	−0.448
retinal_endothelial_cells	0.053	0.379	0.468	−0.170	−0.015	0.414	−0.026	−0.157
ipsc_derived_retinal_organoids	−0.035	0.045	−0.029	−0.122	−0.038	−0.012	−0.056	0.077
trabecular_meshwork_cells	−0.061	−0.046	−0.083	−0.308	−0.118	−0.056	−0.088	−0.230
**Disease**								
Keratoconus	0.858	0.394	0.293	0.390	0.548	−0.538	−0.389	0.461
Age-related macular degeneration	−0.548	−0.545	−0.231	−0.436	−0.522	0.731	0.554	−0.561
Diabetic retinopathy	−0.015	0.167	0.077	0.080	−0.032	−0.308	−0.303	0.176
Primary open-angle glaucoma	0.013	−0.026	−0.099	0.264	0.423	−0.117	−0.219	0.002
Retinitis pigmentosa	−0.032	0.391	0.010	0.025	0.038	−0.102	0.113	0.097
Retinoblastoma	−0.053	0.201	0.076	0.064	−0.027	−0.354	−0.264	0.310

### Example usage 2: integrating GWAS and co-expression network identifies AMD associated module in Eyediseases

**Figure 2.**
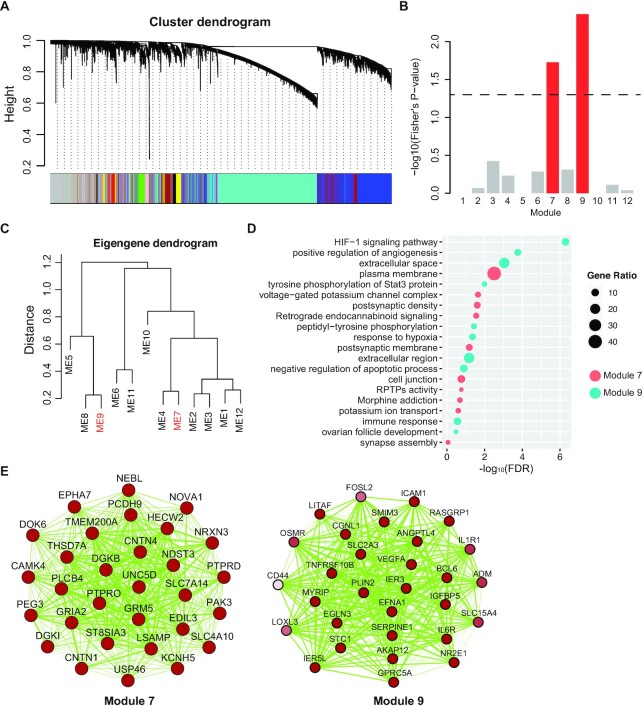
Identification of the AMD Functional Module. (**A**) Weighted gene co-expression network analysis (WGCNA) in retina, showing a hierarchical clustering tree of co-expression modules. Each module corresponds to a branch, which is labeled by a distinct color shown underneath. (**B**) Enrichment of genes located in AMD GWAS regions in network modules 7 and 9. (**C**) Eigengenes for modules 7 and 9 cluster. (**D**) GO enrichment analysis of the module 7 and 9. The rich factor refers to the proportion of enriched genes for each term. (**E**) Top hub genes along with edges supported by co-expression are shown. Hub genes are defined by being the top 30 most connected genes based on kME intermodular connectivity. Increasing edge width indicates increasing topological overlap measure (tom). Increasing color gradient indicates increasing connectivity degree.

Age-related macular degeneration (AMD) is the leading cause of blindness in the elderly with limited therapeutic options (28). Genetic variants can help uncover disease mechanisms and provide entry points into therapy. Since the first AMD GWAS in 2005 (29), genome-wide association studies for AMD have identified dozens of associations; yet, the genes responsible for most associations remain elusive. Genes underlying a disease are often functionally related and functionally related genes are often co-expressed (30). Therefore, we can build a co-expression network of eye tissues to predict associated genes at available GWAS loci of eye diseases. We began by generating a list of 190 genes located within age-related macular degeneration (AMD) GWAS loci from 19 projects in ‘disease-genetics’ function. Next, we constructed co-expression network in R which partitioned into 12 network modules using 107 retina expression profiles from ‘co-expression’ function (Figure 2A). Of the 12 network modules, two (modules 7 and 9) were significantly enriched for AMD associated genes (OR = 4.2, Fisher's *P*-value = 0.01 and OR = 5.1, Fisher's *P*-value = 0.004, respectively) (Figure 2B). To more formally evaluate the relationship between the two modules, we created a network based on the eigengene of each of the 12 modules. The module eigengene corresponds to the first principal component of a given module. In this network, the module 7 and 9 eigengenes were not correlated, which imply AMD have different pathogenesis (Figure 2C).

Next, we evaluated gene ontology (GO) enrichments to determine function of the genes between module 7 and 9 (Figure 2D). Notably, the module 9 was significantly (FDR < 0.05) enriched for Hypoxia-Inducible Factor (HIF)-1 signaling pathway (FDR = 5.04 × 10^−7^) and positive regulation of angiogenesis (FDR = 1.73 × 10^−4^), which have been associated with choroidal neovascularization and the progression of AMD into the neovascular clinical phenotype (nAMD). Regard to nAMD, HIFs regulate the expression of multiple growth factors and cytokines involved in angiogenesis and inflammation, hallmarks of neovascular AMD. HIFs also increase RPE apoptosis and autophagy, similarly to the choriocapillaries, which stimulates the endothelial proliferation observed in nAMD. Additionally, we found that the module 7 was enriched for voltage-gate potassium channel complex (FDR = 0.022) and postsynaptic density (FDR = 0.024), which imply neuronal processes are essential elements in human retinal tissues in response to AMD. Another way to infer the function of the modules is based on the known function of highly connected genes with central positions within the modules (‘‘hub’’ genes). We explored the strongest connections in module 6 and 9 using Cytoscape software (Figure 2E). These results indicated that integrating GWAS and co-expression data, it is possible to provide insight into the identity of associated GWAS genes involved in AMD-related pathways.

## DISCUSSION

EyeDiseases is a first comprehensive database that has been developed for the integration of multiple omics and interactive analysis in eye diseases. The data in EyeDiseases are from GWAS catalog, GWAS Central, OMIM, the GEO database, the EMBL-EBI, ENCODE, TCGA and Drugbank database with thousands of samples included. Data type consisting of the genome, transcriptome, epigenome, single-cell and targeted drug. EyeDiseases is a time-saving, free, and intuitive tool for tapping the full potential of publicly available genomics big data, which enables biologists and clinicians without any programming experience to obtain ready-to-use multi-omics data and perform a diverse range of data analyses. It also has the potential to become a one-stop service for data query and analysis for the scientific and clinical community associated with the eye disorder field.

The majority of genetic variants potentially associated with eye diseases (e.g. AMD) that are identified by GWAS are located in non-coding sequences, suggesting that their functions, if any, may be exerted through regulating the expression of genes producing proteins or non-coding RNAs (31–34). Thus, integration of GWAS and gene expression network is essential for elucidating the underlying mechanisms for causal gene in diseases (35–37). Our Eyediseases database provides integrative data, from which users can explore the functional biological network in both disease tissues and normal eye tissues. As an effective application of Eyediseases to identify potential causal gene for eye disorders, we used an eye-relevant co-expression network to inform GWAS loci robustly associated with AMD and identify putative causal genes. We identified two network modules (7 and 9) that contained more genes implicated by GWAS than would be expected by chance. Modules 7 and 9 were enriched for processes the known mechanisms of AMD, such as ‘Hypoxia-Inducible Factor (HIF)-1 signaling pathway’ (38,39). Based on our results, we believe that significant insight into complex disease etiology can be gained using networks to inform GWAS.

To better serve the research community of eye diseases, we will not only continuously update multi-omics data from both eye disease and normal samples, but also develop new analytical features for further exploration of the available big genomic data. Our next plan is to obtain more public multi-omics data on eye diseases, such CNV, proteomics, ocular image and metagenomics, and build an enhanced database based on comprehensive genome-level data for the effective visualization and analysis of all human genes in the future. We believe Eyediseases could serve as a very useful public resource for both bench scientists and computational biologists, and contribute to clinical and translational studies.
